# P-216. Using an EHR Tool to “Nudge” Earlier *C. difficile* Testing

**DOI:** 10.1093/ofid/ofae631.420

**Published:** 2025-01-29

**Authors:** Carol Briody, Stephen C Eppes, Serena Wingel, Krystal Coles, Edwin Hartman, Crystal Hogate, Lindsay Sanderson, Hayley Sweetser, Shannon Wilton, Marci Drees

**Affiliations:** ChristianaCare, Newark, Delaware; Christiana Care Health System, Newark, DE; ChristianaCare, Newark, Delaware; ChristianaCare, Newark, Delaware; ChristianaCare, Newark, Delaware; ChristianaCare, Newark, Delaware; ChristianaCare, Newark, Delaware; ChristianaCare, Newark, Delaware; ChristianaCare, Newark, Delaware; ChristianaCare, Newark, Delaware

## Abstract

**Background:**

After observing an increase in hospital-onset *Clostridioides difficile* infections (HO-CDI), case review revealed that 50% had community-onset (CO) symptoms, but testing had not been performed until after hospital day 3. An interdisciplinary group convened to determine methods to encourage earlier testing in symptomatic patients and implemented a “Smart Zone Alert” (SZA) in our electronic health record (EHR; Cerner).

**Figure:**
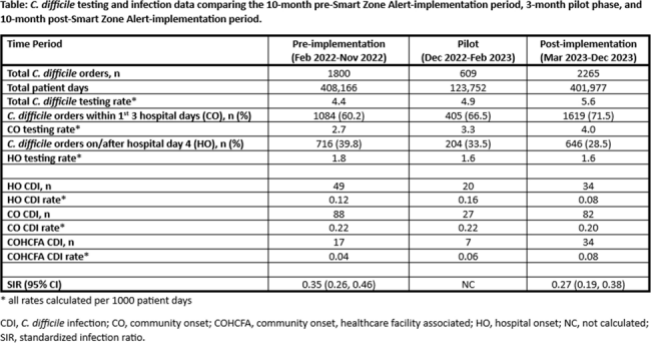

**Methods:**

ChristianaCare is a 3-hospital, > 1200-bed community-based academic health system based in northern Delaware. The SZA is an integrated, real-time notification that encourages frontline providers to order C. diff testing if nursing documents ≥ 3 loose stools/24 hours during the first 3 hospital days. The SZA does not interfere with workflow and can be dismissed or acted upon. The SZA was piloted during Dec 2022 – Feb 2023, initially on 2 but expanding to 12 units at 2 hospitals. A PowerInsight report was built to allow tracking of SZAs and whether testing was ordered. In Mar 2023, the SZA went live across all 3 hospitals. We calculated rates of HO, CO, and community-onset healthcare-facility-associated (COHCFA) CDI for each period (pre-, pilot, and post-SZA) using NHSN definitions, and number and rates of C diff tests obtained before (CO) and after (HO) hospital day 3. We calculated rate-ratios (RR) and standardized infection ratios (SIR) comparing the 10-month pre-SZA-implementation period to the 10-month post-implementation period.

**Results:**

Comparing the pre-SZA and post-SZA periods, the total number of C. diff tests performed increased from 1800 (4.4/1000 pt-days) to 2265 (5.6/1000 pt-days), and the proportion of CO tests increased from 60.2% to 71.5% (RR 1.6, 95% CI 1.5, 1.8) (Table). The HO-CDI rate decreased 30% (RR 0.7, 95% CI 0.45, 1.1), although not statistically significant; while COHCFA-CDI doubled (RR 2.0, 95% CI 1.1, 3.7). As of Dec 2023, the SZA had fired 2876 times and prompted earlier testing that avoided 25 HO-CDI. The SIR decreased from 0.35 (95% CI, 0.26, 0.46) to 0.27 (95% CI 0.19, 0.38).

**Conclusion:**

The SZA is an integrated real-time tool that provides actionable and trackable information about patients’ stooling patterns and encourages earlier C. diff testing, enabling earlier isolation and treatment and avoiding falsely attributing CDI cases as HO.

**Disclosures:**

**All Authors**: No reported disclosures

